# Attributable risk and potential impact of interventions to reduce household air pollution associated with under-five mortality in South Asia

**DOI:** 10.1186/s41256-018-0059-x

**Published:** 2018-01-18

**Authors:** Sabrina Naz, Andrew Page, Kingsley Emwinyore Agho

**Affiliations:** 1 Translational Health Research Institute, School of Medicine, Western Sydney University, Building 3, Campbelltown Campus, Locked Bag 1797, Penrith, NSW 2571 Australia; 2 School of Science and Health, Western Sydney University, Campbelltown Campus, Locked Bag 1797, Penrith, NSW 2571 Australia

**Keywords:** Household air pollution, Under-five mortality, Cooking fuel, Population attributable risk, South Asia

## Abstract

**Background:**

Solid fuel use is the major source of household air pollution (HAP) and accounts for a substantial burden of morbidity and mortality in low and middle income countries. To evaluate and compare childhood mortality attributable to HAP in four South Asian countries.

**Methods:**

A series of Demographic and Health Survey (DHS) datasets for Bangladesh, India, Nepal and Pakistan were used for analysis. Estimates of relative risk and exposure prevalence relating to use of cooking fuel and under-five mortality were used to calculate population attributable fractions (PAFs) for each country. Potential impact fractions (PIFs) were also calculated assessing theoretical scenarios based on published interventions aiming to reduce exposure prevalence.

**Results:**

There are an increased risk of under-five mortality in those exposed to cooking fuel compared to those not exposed in the four South Asian countries (OR = 1.30, 95% CI = 1.07–1.57, *P* = 0.007). Combined PAF estimates for South Asia found that 66% (95% CI: 43.1–81.5%) of the 13,290 estimated cases of under-five mortality was attributable to HAP. Joint PIF estimates (assuming achievable reductions in HAP reported in intervention studies conducted in South Asia) indicates 47% of neonatal and 43% of under-five mortality cases associated with HAP could be avoidable in the four South Asian countries studied.

**Conclusions:**

Elimination of exposure to use of cooking fuel in the household targeting valuable intervention strategies (such as cooking in separate kitchen, improved cook stoves) could reduce substantially under-five mortality in South Asian countries.

## Background

Household air pollution (HAP) from unprocessed fuels traditionally used for cooking is a key public health hazard and one of the leading environmental causes of disability worldwide, predominantly due to respiratory illness and deaths among children under-five years of age in low and lower-middle-income countries [1–3]. In low and middle income countries, burning of solid fuels (such as wood, animal dung, crop residues, charcoal and coal) on traditional stoves or open fire releases harmful health-damaging pollutants/chemicals include carbon monoxide and particulate matter (PM) and exposure to these pollutants result in respiratory illness and deaths [4, 5]. Globally, more than 2.9 million deaths have been attributable to HAP from solid fuel use in 2015 [6]. Women who are responsible for cooking in most developing societies and their accompanying young children are more likely to be exposed to HAP and the development of acute lower respiratory infections (ALRIs) [7–9]. Children have a disproportionate exposure to HAP as air intake of an infant is approximately twice that of adults, exacerbated by time spent indoors, [8] which results in inhaling more pollutants present into the indoor air [10, 11].

South Asia is home to a large, fast-growing population with a substantial proportion of the population living in poverty, and a region of the world with the highest population density [12]. There is a great variation in population size among countries, yet all countries have similar population proportions living in rural areas in poor conditions where access to clean energy resources is limited and more than 80% of the population still rely on solid fuels as domestic source of energy in this region [1, 12, 13]. South Asia has achieved great improvements in population health over the past six decades, and all countries in this region have seen significant declines in under-five mortality (more than a two-thirds decline in infant mortality since the early 1980s) [14]. Substantial progress has been made toward achieving the Millennium Development Goal 4 (MDG 4) target of reducing the under-five mortality rate between 1990 and 2013 from 90 to 46 deaths per 1000 live births, although there are considerable differences between countries and regions [15, 16]. Despite this, ARI remains to be the leading single cause of under-five deaths in South Asia [7, 17]. HAP from the use of solid fuel is mostly associated with ALRIs which is also ranked 7th as a risk factor and 1st as an environmental risk factor in global burden of disease (GBD) study 2013 [18]. As a significant portion of household in South Asia still depend on solid fuels for cooking, understanding the effect of HAP on mortality in children under-five is important. Cooking fuel is a modifiable exposure [19] and eliminating exposure from this risk factor might be a way for further progress toward reducing the burden of under-five mortality.

It is well documented from studies in South Asian countries [20–26] that exposure to HAP from cooking fuel is associated with an increased risk of respiratory infections leading to under-five mortality. However, no previous studies have assessed the burden of under-five mortality that could be attributable by eliminating the exposure from HAP in the population under a more favorable distribution of risk factors based on previous intervention studies in South Asian context. Therefore, the aim of this study was to assess the attributable risk associated with HAP and to examine theoretical scenarios assessing the potential impact of previous interventions [27, 28] to eliminate exposure prevalence associated with under-five mortality in national populations of the four South Asian countries to inform policies and interventions.

## Methods

### Data sources

Nationally representative and publicly available Demographic and Health Survey (DHS) datasets for four South Asian countries such as Bangladesh, India, Nepal and Pakistan were focused in this study, collected with approval from the DHS program (http://www.dhsprogram.com). All existing datasets (for Bangladesh for the years 2004, 2007, 2011 & 2014; for Nepal for 2001, 2006 & 2011; for India for 1992–93, 1998–99 & 2005–06, and for Pakistan for the year 2012–13) with available information on HAP were used in the analysis. The DHS program is responsible for collecting, summarizing and publishing nationally representative data on health and population in developing countries. This program is implemented by ICF international of Calverton, Maryland, USA and financial support from the United States Agency for International Development (USAID). Demographic and heath data of urban and rural areas were collected by interviewing ever-married women and men using a stratified sample of households based on a two-stage cluster design with an average response rate of more than 90% in South Asia. An index period of five years was used for each survey to minimize recall bias of child birth and death information which was self-reported by the mother. A total of 221,745 singleton live-born children from Bangladesh, India, Nepal and Pakistan were obtained in this study, of which 13,290 died within 5-years prior to the mother being interviewed.

### Study outcomes

The main outcome variable in this study was under-five mortality (number of deaths between birth and the fifth birthday, 0–59 months). The analyses were also carried out for neonatal mortality (number of deaths during the first 28 days of life, 0–28 days), as most of the deaths occurred in the first month of life of children in these study countries. Outcome variables were considered dichotomous for the analysis.

### Exposure to cooking fuel

The main exposure of interest was the type of cooking fuel used in the household. The respondents were asked, “What type of fuel does your household mainly use for cooking?” The reported fuels were categorised into “clean fuels” (electricity, liquid petroleum gas (LPG), natural gas and biogas) and “polluting fuels” (kerosene, coal/lignite, charcoal, wood, straw/shrubs/grass, agricultural crop and animal dung) in the analysis. Previous studies reporting the adverse effects of exposure to kerosene fuel has raised questions about the magnitude of the effect of HAP on under-five mortality or respiratory illness when kerosene has been categorized as a clean fuel [20, 29, 30]. Therefore, kerosene was categorised in the polluting fuels group in this study. A major portion of households in Bangladesh (around 80%), India (around 55%), Nepal (around 75%) and Pakistan (around 60%) use wood/animal dung/crop residues for cooking and a small portion of households in Bangladesh (around 2%), India (around 5%), Nepal (around 4%) and Pakistan (around 3%) use charcoal/coal/kerosene for cooking according to the recent DHS datasets used for analysis in these four countries [31–34]. This study aggregated broad classification for type of cooking fuel to ensure consistent comparison across countries, and also because dis-aggregation of the HAP variable into the sub-groups resulted in small numbers of cases of under-five mortality, and insufficient numbers in some fuel types, for meaningful analysis in multivariate models.

### Potential confounders

For investigating the association between use of cooking fuel and under-five mortality, a number of potential confounding factors were defined for each study country comprising place of residence, wealth index of household, mother’s age, mother’s education, mother’s working status, breastfeeding status of mother, sex of child, location of kitchen, type of house and wall and floor material of household. These socio-economic, individual and behavioural factors have previously been identified as potential confounders for the association between HAP and under-five mortality in developing countries [20, 25, 26, 35–41]. In the analysis, the household wealth index was calculated using principal components analysis to create a country-specific variable that placed households into quintiles based on household assets such as ownership of transport and durable goods, materials for housing construction and facilities in the household [42–44]. Inclusion of these confounding factors varied slightly for each country on the basis of availability of information in DHS datasets.

### Statistical analysis

Incidence proportions of neonatal and under-five mortality were calculated by following the similar method provided in the guideline of the DHS program [45]. Prevalence estimates and 95% of confidence intervals (CI) were calculated to adjust for the cluster sampling survey design by using the “svy” command. For estimates of relative risk between exposure groups, random effects multilevel logistic regression models were conducted using the “xtlogit” function adjusted for potential confounders identified for each country. Summary relative risk estimates for the four South Asian countries were calculated using random effects meta-analysis following the inverse variance method (DerSimonian-Laird) by using the “metan” function. Analyses were carried out in STATA version 13.1 (Stata Corp: College Station, TX, USA). In addition, summary prevalence estimates for the four South Asian countries were calculated using random effects meta-analysis using the MetaXL [46].

The population attributable fraction (PAF) was used to measure the proportion of neonatal and under-five mortality in South Asia that could theoretically be prevented by eliminating HAP from the use of cooking fuels in households, assuming the other risk factors remain unchanged. The PAF was calculated by using the following formula [47, 48],

$$ \mathrm{PAF}=\frac{\sum p\left(\mathrm{RR}-1\right)}{\left(1+\sum p\left(\mathrm{RR}-1\right)\right)} $$

Where *p* is the prevalence of exposure from use of cooking fuel and RR is the adjusted relative risk estimated from DHS datasets for Bangladesh, India, Nepal and Pakistan. A joint PAF across four South Asian countries was also calculated by using the following formula [48, 49],

$$ \mathrm{PAF}\ \left(\mathrm{Combined}\right)=1-\prod \limits_{r=1}^R\left(1-{\mathrm{PAF}}_r\right) $$

Where *r* denotes each individual risk factor. PAF assumes that risk factors are independent and uncorrelated and has been reduced by the use of relative risks that has been adjusted for potential confounders.

However, PAF provides estimates based on the unrealistic counterfactual scenario of the elimination of an exposure from a given population (that is, a reduction of the population prevalence to zero) [50]. This estimate assumes that 100% of the exposed population could switch to clean fuels from polluting fuels. Population impact fractions (PIF) estimate potential improvements in mortality and burden of the disease under theoretical counterfactual scenarios that could reasonably be achieved in a population [51, 52]. This study also considered PIF estimates for comparison with PAF estimates to measure the theoretical minimum prevalence for exposure in each study country by applying the evidence-base of the potential impact on reductions in HAP from previous interventions [28]. The PIF was calculated by using the following formula [53],

$$ \mathrm{Potential}\kern0.17em \mathrm{Impact}\kern0.17em \mathrm{Fraction}\;\left(\mathrm{PIF}\right)=\frac{\sum_{c=1}^n{p}_c{RR}_c-{\sum}_{c=1}^n{p}_c^{\ast }{RR}_c}{\sum_{c=1}^n{p}_c{RR}_c} $$

Where *p*_*c*_ is the proportion of the neonatal and under-five children in a given exposure category *c*, *RR*_*c*_ is the adjusted relative risk for that category estimated from the DHS datasets and *p*_c_^*^ is the estimated proportion in category *c* after the intervention. In the analysis for this present study, the estimated effect of interventions was based on previously published intervention studies in South Asia [27, 28]. The first scenario assumed a 22% reduction in HAP based on the effect size of a community-based intervention study in Pakistan that relocated the kitchen from indoors to outdoors [27]. A second scenario assumed a 63% reduction in HAP based on the effect size of a community-based study in Nepal that involved improvements in cooking stoves by installing ventilated chimneys [28]. Estimated exposure prevalence both before and after interventions for each country are presented in Table 1.

**Table 1:** Relative risk, exposure prevalence for PAF, PIF and cases of neonatal and under-five mortality due to HAP in the four South Asian countries

	Country	Exposure^a^	Exposure (%) in cases (n/Total cases)^b^	Exposure (%) in controls (n/Total controls)^c^	Relative risk (95% CI)^d^	Exposure Prevalence (95% CI)^e^	Estimated Prevalence (scenario 1) (95% CI)^f^	Estimated Prevalence (scenario 2) (95% CI)^g^
Neonatal mortality	Bangladesh	No HAP from cooking fuel	63/772	2773/25304	1.00	11.0 (10.6–11.4)	30.6 (30.0–31.1)	67.1 (66.5–67.6)
		HAP from cooking fuel	709/772	22,531/25304	1.29 (0.94–1.78)	89.0 (88.7–89.4)	69.5 (68.9–70.0)	33.0 (32.4–33.5)
	India	No HAP from cooking fuel	565/6082	26,783/160300	1.00	16.7 (16.5–16.9)	35.0 (34.8–35.3)	69.2 (69.0–69.4)
		HAP from cooking fuel	5517/6082	133,517/160300	1.23 (1.09–1.39)	83.3 (83.1–83.5)	65.0 (64.7–65.2)	30.8 (30.6–31.0)
	Nepal	No HAP from cooking fuel	15/572	1303/17208	1.00	7.6 (7.2–8.0)	27.9 (27.2–28.6)	65.8 (65.1–66.5)
		HAP from cooking fuel	557/572	15,905/17208	2.67 (1.47–4.82)	92.4 (92.0–92.8)	71.1 (71.4–72.8)	34.2 (33.5–43.9)
	Pakistan	No HAP from cooking fuel	136/503	3909/11004	1.00	35.5 (34.6–36.4)	49.7 (48.8–50.7)	76.1 (75.3–76.9)
		HAP from cooking fuel	367/503	7095/11004	1.08 (0.77–1.54)	64.5 (63.6–65.4)	50.3 (49.4–51.2)	23.9 (23.1–24.7)
	Total^h^		7929	213,816	1.32 (1.05–1.67)	83.8 (75.2–90.9)		
Under-five mortality	Bangladesh	No HAP from cooking fuel	107/1211	2729/24865	1.00	11.0 (10.6–11.4)	30.6 (30.0–31.1)	67.1 (66.5–67.7)
		HAP from cooking fuel	1104/1211	22,136/24865	1.06 (0.82–1.37)	89.0 (88.6–89.4)	69.4 (68.9–70.0)	32.9 (32.4–33.5)
	India	No HAP from cooking fuel	850/11311	26,498/155071	1.00	17.1 (16.9–17.3)	35.3 (35.1–35.6)	69.3 (69.1–69.6)
		HAP from cooking fuel	10,461/11311	128,573/155071	1.30 (1.18–1.43)	82.9 (82.7–83.1)	64.7 (64.4–64.9)	30.7 (30.5–30.9)
	Nepal	No HAP from cooking fuel	25/1014	1293/16766	1.00	7.7 (7.3–8.1)	28.0 (27.3–28.7)	65.9 (65.1–66.6)
		HAP from cooking fuel	989/1014	15,473/16766	2.19 (1.37–3.51)	92.3 (91.9–92.7)	72.0 (71.3–72.7)	34.2 (33.4–34.9)
	Pakistan	No HAP from cooking fuel	197/768	3848/10739	1.00	35.8 (34.9–36.8)	50.0 (49.0–50.9)	76.3 (75.4–77.1)
		HAP from cooking fuel	571/768	6891/10739	1.22 (0.92–1.64)	64.2 (63.3–65.1)	50.1 (49.1–51.0)	23.7 (22.9–24.6)
	Total^h^		13,290	207,441	1.30 (1.07–1.57)	83.6 (74.9–90.8)		

^a^Exposure were categorised as, “no HAP from cooking fuel” (use of clean fuels such as electricity, LPG, natural gas, biogas) and “HAP from cooking fuel” (use of polluting fuels such as kerosene, coal/lignite, charcoal, wood, straw/shrubs/grass, agricultural crop and animal dung), ^b^number of ‘cases’ exposed/unexposed in each category, ^c^number of ‘controls’ exposed/unexposed in each category, ^d^Relative risk adjusted for selected potential confounders, *PAF* Population attributable fraction, *PIF* Potential impact fraction, ^e^exposure prevalence for PAF calculation, ^f^exposure prevalence for PIF calculation based on scenario1: assuming 22% reduction on HAP following previous intervention [27], ^g^exposure prevalence for PIF calculation based on scenario2: assuming 63% reduction on HAP following previous community-based intervention [28]. ^h^Total number of cases and controls were summed across country-specific DHS data sets, and Relative Risk and exposure prevalence were summarised across countries using random effects meta-analysis using the inverse variance method (DerSimonian-Laird)

Monte-Carlo simulation models were used to estimate the 95% confidence intervals (CI) for PAF and PIF estimates, to account for the uncertainty around the exposure prevalence and relative risk estimates using the Ersatz Software1.31 in MS Excel [54]. A beta probability distribution was used to estimate exposure prevalence (based on cases and controls) using the ErBeta function and a normal distribution was used to estimate relative risk (based on a normal distribution for the natural logarithm of the RR) using the ErRelative function to calculate 95% CI for PAF and PIF estimates after 10,000 iterations to ensure convergence of the model. The PAF and PIF estimates were then multiplied by the estimated total number of mortality cases to obtain the estimated number of neonatal and under-five mortality cases attributable to each study country.

## Results

The overall pooled relative risk estimates in the four South Asian countries suggested a significant association between use of cooking fuel and under-five mortality (OR = 1.30, 95% CI = 1.07–1.57, *P* = 0.007) and neonatal mortality (OR = 1.32, 95% CI = 1.05–1.67, *P* = 0.019) (Table 1). Of the 13,290 estimated cases of under-five mortality in the four South Asian countries, 3.9% (95% CI: -20.8-24.9%) from Bangladesh (1211 cases), 19.8% (95% CI: 12.9–26.3%) from India (11,311 cases), 49.8% (95% CI: 23.5–69.5%) from Nepal (1014 cases) and 11.4% (95% CI: -6.9-29.0%) from Pakistan (768 cases) were attributable to HAP from use of cooking fuel (Table 2). The joint PAF associated with HAP for the four South Asian countries was 65.7% (95% CI: 43.1–81.5%) for all cases of under-five mortality and 71.4% (95% CI: 46.0–86.9%) for all cases of neonatal mortality (Table 2).

**Table 2:** PAF and PIF estimates for neonatal and under-five mortality associated with HAP in the four South Asian countries

	Country	Cases^a^	PAF% (95% CI)^b^	PIF% (95% CI) (scenario 1 [27])^c^	PIF% (95% CI) (scenario 2 [28])^d^
Neonatal mortality	Bangladesh	772	18.5 (−8.1–40.2)	4.1 (−1.7–8.9)	11.6 (−4.9–25.5)
	India	6082	15.8 (6.8–24.2)	3.5 (1.4–5.9)	10.0 (4.3–15.4)
	Nepal	572	56.8 (25.7–77.7)	12.5 (5.4–17.1)	32.3 (10.3–47.0)
	Pakistan	503	3.5 (−19.2–25.0)	0.8 (−4.6–5.9)	2.3 (−12.5–15.7)
	Joint PAF/PIF	–	71.4 (46.0–86.9)	19.6 (10.4–27.2)	47.4 (25.3–63.1)
	Total	7929	–	–	–
Under-five mortality	Bangladesh	1211	3.9 (−20.8–24.9)	2.1 (−3.6–6.9)	6.0 (−9.6–19.5)
	India	11,311	19.8 (12.9–26.3)	4.3 (2.8–5.8)	12.5 (8.3–16.6)
	Nepal	1014	49.8 (23.5–69.5)	10.9 (4.7–15.4)	27.6 (8.6–41.6)
	Pakistan	768	11.4 (−6.9–29.0)	1.3 (−2.9–5.2)	3.7 (−7.0–13.8)
	Joint PAF/PIF	–	65.7 (43.1–81.5)	17.6 (9.6–24.6)	42.7 (22.8–57.6)
	Total	13,290	–	–	–

^a^Total number of neonatal and under-five mortality cases associated with use of cooking fuel, ^b^Population attributable faction (PAF) estimates attributable to HAP, ^c^Potential impact faction (PIF) estimates based on 22% reduction on HAP, ^d^Potential impact faction (PIF) estimates based on 63% reduction on HAP

Potential impact fraction estimates in the four South Asian countries assuming a 22% reduction on HAP [27] found that HAP was associated with 2.1% (95% CI: -3.6-6.9%) of under-five mortality in Bangladesh, 4.3% (95% CI: 2.8–5.8%) in India, 10.9% (95% CI: 4.7–15.4%) in Nepal and 1.3% (95% CI: -2.9-5.2%) in Pakistan (Table 2). In comparison, impact fractions assuming a larger effect size of a 63% reduction on HAP [28] found that HAP was associated with 6.0% (95% CI: -9.6-19.5%) of under-five mortality for Bangladesh, 12.5% (95% CI: 8.3–16.6%) for India, 27.6% (95% CI: 8.6–41.6%) for Nepal, and 3.7% (95% CI: -7.0-13.8%) for Pakistan (Table 2).

## Discussion

This study estimated the attributable burden of under-five mortality associated with the use of cooking fuel in the four South Asian countries. The summary association for the four South Asian countries found that the risk of under-five mortality was approximately 30% higher in the polluting fuel group compared to clean fuel group. Findings suggested that more than 65% of cases of under-five mortality were attributable to cooking fuel. However, estimates of attributable burden based on theoretical minimum prevalence of HAP exposure suggested that between 18% and 43% of under-five mortality cases were avoidable assuming that the effects of previous intervention studies that re-locate kitchens and improve ventilation of cooking stoves [27, 28] could be scaled to South Asian populations.

Studies from South Asia [20–26] have consistently acknowledged the risk of under-five mortality from use of cooking fuel. This study estimated the proportion of under-five mortality in Bangladesh, India, Nepal and Pakistan that could, in combination, be attributable from exposure to use of polluting fuels for cooking. This study found that Nepalese children were in higher risk of neonatal and under-five mortality than in India, Bangladesh and Pakistan. A recent DHS report from Nepal indicated that more than 70% of the houses in rural areas were constructed using wood and mud with poor ventilation, and use of solid fuels (approximately 82%) for cooking was very common in rural residence and 71% of Nepalese households did not have any separate kitchen [32]. Similarly, an average of 80% of rural households in Bangladesh, India and Pakistan still depend on solid fuels for cooking [1]. In addition, more than 90% of Pakistani households, 30% of Indian and 15% of Bangladeshi households did not have any separate kitchen in the house for cooking according to the recent DHS datasets [31–34]. This study also showed the risk of deaths was slightly higher in the neonatal period than in older children in the four South Asian countries. This is biologically plausible as children’s metabolic pathways, specifically in the first month after birth is unformed and less able to deal with toxic chemicals from polluting fuels; hence young children are more vulnerable to these pollutants [10]. However, we found the stronger effect in under-five mortality than neonatal mortality associated with the use of cooking fuel for our study countries of India and Pakistan, which may indicate the possibility of cumulative effects over the early years of childhood in these contexts.

The findings from the present study suggest that switching entirely to cleaner fuels (such as electricity, liquid petroleum gas (LPG), natural gas and biogas) from polluting fuels could reduce more than 65% of cases of under-five mortality. However, in practice, not all poor rural and urban communities could feasibly eliminate their use of solid fuels [21, 22, 27, 55–57]. Moreover, access to clean alternatives is limited due to availability and affordability, and thus solid fuels remain the most practical fuel in poor families of South Asian countries [21, 22, 27, 55–57]. To provide increased access to clean fuels in neglected communities, a long term strategy and government investment programs are required; thus, shorter term, more efficient, cost-effective, and sustainable policies need to be promoted in South Asia. Studies from developing countries [9, 21–24] have shown a higher risk of under-five mortality in those households using solid fuels in inside kitchens than in houses with separate kitchens. A community-based intervention study in Pakistan suggested that improved design of the cooking space or relocating the kitchen outside instead of inside the house could reduce particulate matter (PM) concentrations by 22% [27]. Assuming this 22% of reduction to calculate PIFs, this study suggested approximately 2%, 4%, 11% and 1% of cases of under-five mortality in Bangladesh, India, Nepal and Pakistan respectively, could be avoided.

Another sustainable alternative of this problem is to use improved cooking stoves with chimney installation (typically made of clay and husk) proposed in previous intervention reports and studies [28, 58–60]. Improved cooking stoves when used properly have been found to be energy efficient as well as beneficial for health as they reduce emissions of particulate matter (PM) [61], and the United Nation Foundation’s Global Alliance for clean cooking stoves announced the goal to make 100 million homes to adopt clean stoves by 2020 [62]. The recent RESPIRE trial on Guatemala suggested that improved cooking stoves might reduce exposure to HAP by 50% [63]. Similarly, and intervention study from Nepal also found that a 63% of reduction on PM concentrations could be achieved by using improved cooking stoves [28]. Under scenarios which applying these reductions in exposure to HAP in the present study, approximately 6%, 13%, 28% and 4% of all cases of under-five mortality associated with use of cooking fuel could be avoided in Bangladesh, India, Nepal, and Pakistan respectively.

Women’s empowerment is another important factor for decreasing under-five mortality, as women are responsible for cooking in most of the South Asian countries [8, 9, 57, 59, 62]. Mothers and their young children are the main household members who frequently are exposed to cooking smoke as they spend long hours on cooking and disproportionately be affected on related health outcomes [8, 9]. Educating mothers about health risk of using polluting fuels and benefits of cooking in a separate kitchen with improved stoves and the need to keep children away from cooking area could reduce the risk of deaths of young children.

This present study has a number of limitations and strengths. Firstly, categorising study participants into exposed and non-exposed to cooking fuel may lead to misclassification bias, for example, some households use a combination of clean and polluting fuels even where clean fuels are available to cut down their fuel expenditure using solid fuels which are cheap and affordable option in South Asian context [57, 64–66]. However, DHS program is only collected information on use of primary fuel in the household. Moreover, even within the same type of cooking fuel (for example, wood is the most common type of solid fuel used for cooking in these four countries [31–34]), the HAP concentrations and exposure level might vary largely due to different household settings and personal behaviour across countries. For instance, very few households (only 15%) in Bangladesh did not have any separate kitchen for cooking where in Pakistan more than 90% and in Nepal more than 70% of households did not have any separate kitchen for cooking [32–34]. Previous studies found that use of polluting fuels for cooking in indoor without a separate kitchen increased the risk of under-five mortality [21–24, 39]. Secondly, birth and death information on children were self-reported by respondents that may be source of recall bias, however this study restricted their analysis to those children born within five years preceding the survey to reduce the likelihood of this bias. Thirdly, this study considered all-cause mortality for this analysis in the absence of information on cause-specific deaths in the DHS surveys, and is likely to be a source of ascertainment bias in mortality outcomes. Lastly, these studies used a series of cross-sectional datasets for analysis; and as such, clear temporal associations between exposure (use of cooking fuel) and outcome (under-five mortality) cannot be clearly defined when collected at the same point of time. Actual levels and patterns of exposure to emission from cooking smoke were not measured in this study due to the absence of such objective measures in DHS survey.

Despite these methodological limitations, this study incorporated large, nationally representative datasets from Bangladesh, India, Nepal and Pakistan with a very high response rate (approximately 90%) from participants. A number of international studies from many countries including GBD study [7, 18, 48, 67–69] used similar methods to assess attributable burden to identify the level of preventable disease and to implement potential health research. However, this is the first study that has assessed the attributable or avoidable burden of under-five mortality cases associated with HAP from cooking fuel based on potential impact of previous community-based interventions. Despite the fact that under-five mortality in South Asia has dropped over the past decades, more than 80% of rural and semi-urban households still rely on solid fuels for cooking [12, 70], therefore HAP remains a common and modifiable exposure in the population and a key public health priority for Bangladesh, India, Nepal and Pakistan. Findings suggested that more than 65% of cases of under-five mortality were attributable to HAP by switching entirely to clean fuels. In addition, this study also investigated the scenarios of potential impact of previous interventions (such as separate kitchen, improved cooking stoves) to prevent burden of under-five mortality associated with use of polluting fuels that will be more appropriate approach and helpful for health care policy makers to implement preventive strategies in the four South Asian countries.

## Conclusions

The burden of under-five mortality associated with HAP in South Asia remains a significant and preventable public health problem. The wider implementation of programs targeting the modifiable exposure of cooking fuel, through improvements in house design, behavioural intervention, health system policies and continued economic development particularly rural and low income urban communities has the potential to substantially reduce child mortality burden in South Asia.

## Abbreviations

ALRIs: Acute Lower Respiratory Infections
DHS: Demography and Health Survey
GBD: Global Burden of Disease
HAP: Household Air Pollution
LPG: Liquid Petroleum Gas
MDG: Millennium Development Goal
PAF: Population Attributable Fraction
PIF: Potential Impact Fraction
USAID: United States Agency for International Development
WHO: World Health Organization
